# Observational Study on Progressive Muscle Relaxation and Breathing Control for Reducing Dental Anxiety in Children

**DOI:** 10.3390/medicina61050876

**Published:** 2025-05-10

**Authors:** Sorana Maria Bucur, Ioana Maria Crișan, Dorin Ioan Cocoș, Eugen Silviu Bud, Carmen Galea

**Affiliations:** 1 Department of Dentistry, Faculty of Medicine, Dimitrie Cantemir University of Târgu Mureș, 540545 Târgu Mureș, Romania; bucursoranamaria@gmail.com (S.M.B.); unicapharm@gmail.com (C.G.); 2 Faculty of Medicine, George Emil Palade University of Medicine, Pharmacy, Science, and Technology of Târgu Mures, 38 Ghe. Marinescu Street, 540142 Târgu Mures, Romania; 3 Faculty of Dental Medicine, “Dunărea de Jos” University, 800008 Galați, Romania; 4 Department of Orthodontics, George Emil Palade University of Medicine, Pharmacy, Science, and Technology of Târgu Mures, 38 Ghe. Marinescu Street, 540142 Târgu Mures, Romania; eugen.bud@umfst.ro

**Keywords:** dental anxiety, youth, Jacobson’s progressive muscle relaxation, breathing control, physiological stress, IDAF-4C^+^

## Abstract

*Background and Objectives*: Dental anxiety is a common barrier to pediatric oral care. Non-pharmacological relaxation techniques like Jacobson’s Progressive Muscle Relaxation (JPMR) and Breathing Control (BC) may help reduce psychological and physiological stress. This study assessed the utility of JPMR and BC in reducing dental anxiety and physiological arousal in children and adolescents. *Materials and Methods*: In this observational study, 189 participants aged 8–17 undergoing non-invasive dental procedures were assigned to JPMR (n = 63), BC (n = 63), or control (n = 63) groups. Dental anxiety was measured with the Romanian-validated IDAF-4C^+^, and physiological stress was measured via blood pressure and heart rate. Pre and post-intervention data were analyzed using paired *t*-tests, ANOVA, and cluster analysis. *Results*: JPMR led to the highest reductions in IDAF-4C^+^ scores (Δ = −1.23, *p* < 0.001, *d* = 1.12) and systolic blood pressure (Δ = −9.4 mmHg, *p* < 0.01). BC showed moderate anxiety reduction (Δ = −0.64, *p* < 0.05, *d* = 0.61) with minor physiological changes. The control group showed no significant change. Cluster analysis revealed three response patterns: (1) high anxiety–strong responders (n = 58), mainly benefiting from JPMR; (2) moderate anxiety–partial responders (n = 74); and (3) low anxiety–non-responders (n = 57). Younger age and female gender were linked to better JPMR response. *Conclusions*: JPMR is an effective and practical method for reducing dental anxiety and physiological stress in pediatric dental care.

## 1. Introduction

Dental anxiety (DA) is an emotion similar to fear but diffuse, anticipatory, and nonspecific, manifested in the dental office. It is essential to differentiate between dental fear, dental phobia, and dental anxiety, which are three different ways of perceiving dental treatment and lead to three different types of reactions to this medical intervention [1]. Dental fear is a specific fear reaction toward a particular object or clearly defined situation within the context of dental treatment [2]. The distinction between dental anxiety and dental fear is difficult to make [1,2]. Dental phobia goes beyond fear or anxiety, leading to an avoidance behavior toward treatment, which the patient perceives as a disproportionately large threat compared to the actual situation [3].

A systematic review with meta-analysis [4] found that the overall estimated prevalence of DA was 23.9%, with a 95% confidence interval ranging from 20.4% to 27.3%. When broken down by age group, the pooled prevalence was highest among preschool-aged children at 36.5%, followed by school-aged children at 25.8% (95% CI: 19.5–32.1) and adolescents at 13.3% (95% CI: 9.5–17.0). A higher incidence of DA was observed in preschoolers and school-aged children with a history of dental caries and in adolescent girls.

A systematic review [5] found that approximately 30% of children aged 2–6 exhibit dental fear and anxiety (CI: 25–36%). Dental anxiety (DA) was significantly more likely in children without prior dental visits (OR: 1.37; 95% CI: 1.18–1.59) and in those with a history of caries (OR: 1.18; 95% CI: 1.09–1.27). Baier et al. [6] reported that 20% of children experience DA, with 21% displaying negative behavior during treatment, behaviors often correlating with higher anxiety levels. Klingberg and Broberg [7] observed DA in 10% of children and adolescents, with prevalence ranging from 5.7% to 19.5%, influenced by demographic and environmental factors. DA is more common in females and younger children.

Anxiety is a temporary emotional state that varies according to individual characteristics, health, mood, fatigue, and family environment [8]. It can arise from temperament and past negative experiences, reflecting a combination of innate and learned factors [8,9]. Children demonstrate coping behaviors; some remain cooperative using emotion-focused strategies, while others exhibit heightened reactivity and more exaggerated responses [10].

Physiologically, anxiety activates the sympathetic nervous system, triggering responses like hyperventilation, respiratory alkalosis, dyspnea, and sensations of choking [11,12,13]. Cardiovascular effects include elevated blood pressure and pulse rate [14]. These physiological markers are used in studies to assess emotional arousal and the impact of behavioral interventions such as relaxation techniques [15]. Normal pediatric blood pressure ranges vary by age, sex, and height percentile, as shown in Table 1 [16].

Heart rate (HR) values for children and adolescents, according to Kliegman et al. [16], are illustrated in Table 2:

Clinical assessments must consider height, fitness level, stress, and medications. Reference values for vital signs are averages, not absolute diagnostic thresholds. For instance, athletic adolescents may exhibit resting heart rates as low as 40–50 bpm. In contrast, anxiety, elevated catecholamines, or hyperthyroidism can increase heart rate [16]. These values reflect a resting state; heart rate naturally rises with physical or emotional stress.

Over the past decade, non-pharmacological approaches for managing dental anxiety (DA) have gained increasing attention. Relaxation techniques offer safe, low-cost alternatives to anxiolytics like benzodiazepines, which can cause adverse effects [17]. Clinicians are encouraged to use the least invasive strategies to manage anxiety.

This study employed two validated methods: Breathing Control (BC) [18,19,20,21,22,23] and Jacobson’s Progressive Muscle Relaxation (JPM) [3,18,19]. Both were selected for their simplicity, accessibility, and clinical utility in pediatric dental care.

BC is especially effective during acute anxiety or panic episodes. It aims to rebalance autonomic activity, reversing the hyperventilation-induced CO_2_ drop that can exacerbate panic symptoms [19,20,21]. The child is seated comfortably, guided to inhale through the nose for 3–4 s and exhale calmly for 4 s, promoting diaphragmatic breathing and parasympathetic activation [21,23]. Sessions last 5–8 min, often accompanied by soft speech and music. Clinical evidence supports BC’s effectiveness in reducing pediatric dental anxiety; Peretz and Gluck [22] found significant reductions in anxiety using structured breathing techniques [23].

JPM, developed by Edmund Jacobson, involves sequential tensing and relaxing of muscle groups to induce full-body relaxation [3]. Muscle groups are tensed for 6–10 s, then relaxed for 15–20 s, heightening awareness of tension–release contrasts. Typically learned in ~15 min, JPM reduces muscle tension and anxiety-related physiological responses (Appendix A) [3].

JPM functions via both top-down (cortical initiation of movement) and bottom-up (sensory feedback triggering central relaxation) mechanisms [17,18,19]. This dual pathway enhances stress relief and emotional regulation.

BC and JPM are non-invasive, easily implemented in pediatric dental settings, and contribute to improved cooperation and treatment outcomes.

The Index of Dental Anxiety and Fear (IDAF-4C^+^), developed by Armfield [24], is a standardized instrument for assessing dental fear and anxiety. It includes three components: a core module evaluating cognitive, behavioral, emotional, and physiological responses via a 5-point Likert scale (1 = strongly disagree; 5 = strongly agree); a stimuli section measuring anxiety across ten dental-related scenarios; and a phobia module comprising five yes/no items to identify potential dental phobia.

Scoring is based on the core component, with mean scores ≥ 3 indicating the presence of dental anxiety and scores < 3 suggesting minimal or no anxiety [24,25].

A validated Romanian version of the IDAF-4C^+^ [26] demonstrated strong psychometric properties in a 2023 study, showing high internal consistency (Cronbach’s α = 0.945) [27] and strong convergent validity with the Dental Anxiety Scale and a single-item fear measure (Appendix B). This confirms its suitability for use in Romanian populations. This study investigated the effectiveness of two non-pharmacological relaxation techniques, BC and JPM, on dental anxiety. Outcomes were assessed using psychological data from the IDAF-4C^+^ and physiological indicators such as blood pressure and heart rate.

The null hypothesis (H_0_) stated that BC, JPM, and the control group would not differ significantly in their effects on reducing dental anxiety.

## 2. Materials and Methods

### 2.1. Study Design

This observational study examined the associations between two psychological relaxation techniques, Breathing Control (BC) and Jacobson’s Progressive Muscle Relaxation (JPMR), and the levels of dental anxiety and physiological stress in children and adolescents. The study was conducted at the Dimitrie Cantemir University’s Dental Clinic between December 2024 and March 2025.

Participants were observed in three naturally formed groups:

- Group A (Breathing Control) practiced guided diaphragmatic breathing before dental treatment.
- Group B (JPMR) received a brief structured muscle relaxation session before treatment.
- The Control Group received standard dental care with no relaxation intervention.

### 2.2. Participants

Inclusion Criteria

- Children and adolescents aged 8–17 years;
- Scheduled for non-invasive prophylactic dental procedures;
- Fluent in Romanian or able to follow instructions with minimal support;
- Provided informed consent by parent/guardian and assent by the child.

Exclusion Criteria

- Diagnosed cognitive or developmental disorders;
- Current psychiatric treatment, anxiolytic medication use;
- Acute dental pain or emergency treatment needs.

### 2.3. Ethical Considerations

This study was conducted in accordance with the Declaration of Helsinki and approved by the Ethics Committee of “Dunărea de Jos” University (No. 16/CEU/2024, 9 December 2024). Participation was voluntary, and informed consent was obtained from all guardians, with assent from the minors.

### 2.4. Sample Size Calculation and Group Allocation

A priori power analysis was performed using G*Power (version 3.1.9.7) [28] for a one-way ANOVA with three groups. Assuming a medium effect size (*f* = 0.25), significance level α = 0.05, and power (1–β) = 0.80, the minimum required total sample size was 159 participants (53 per group). To ensure sufficient power and to account for possible dropouts, 189 participants were enrolled and evenly distributed across the three groups:

- Group A (Breathing Control): n = 63;
- Group B (JPMR): n = 63;
- Control Group: n = 63.

This exceeded the calculated minimum, providing adequate statistical power (>80%) to detect significant between-group differences.

Participants were allocated to groups based on scheduling availability and clinic flow, ensuring balanced demographic distribution. Due to the nature of the interventions, neither participants nor providers were blinded. However, outcome assessments were conducted by trained, independent evaluators following standardized protocols.

### 2.5. Psychological Measures

Dental Anxiety was measured using the Romanian-validated version of the Index of Dental Anxiety and Fear (IDAF-4C^+^) [26] with the following:

- Core module (8 items) that evaluates emotional, behavioral, cognitive, and physiological anxiety dimensions using a 5-point Likert scale.
- Interpretation: A mean score ≥ 3 indicates clinically significant dental anxiety.

The Romanian version of the IDAF-4C^+^ used in this study was previously validated, demonstrating strong internal consistency (Cronbach’s α = 0.945) and convergent validity with established dental anxiety scales [26]. In the present study, we re-assessed internal consistency using Cronbach’s alpha, with α ≥ 0.70 considered acceptable.

### 2.6. Physiological Measures

Blood Pressure (BP) and Heart Rate (HR) were measured in resting conditions using a validated pediatric automatic monitor (Omron HEM-907XL, Omron Healthcare Co., Ltd., Kyoto, Japan), which is commonly utilized in clinical settings and approved for pediatrics:

- Baseline: Measured before intervention or procedure;
- Post-intervention: Measured immediately after the dental treatment.

### 2.7. Intervention Protocols

- Group A (BC): A 5 min diaphragmatic breathing session guided by trained personnel; participants were instructed to inhale deeply through the nose and exhale slowly through the mouth, with hand-on-abdomen for tactile feedback.
- Group B (JPMR): A 10 min JPMR session guided by audio and an assistant, focused on sequential tensing and relaxing muscle groups (hands, arms, shoulders, face, legs), emphasizing the contrast between tension and relaxation.
- The Control Group received routine dental care without any relaxation techniques.

### 2.8. Procedure Overview

All participants began with an initial evaluation that involved completing the IDAF-4C^+^ questionnaire and baseline blood pressure (BP) and heart rate (HR). They were then divided into three groups: Group A and Group B received specific relaxation interventions—Breathing Control and Jacobson’s Progressive Muscle Relaxation, respectively—while the control group proceeded directly to dental treatment without any intervention. After the treatment, all participants completed the IDAF-4C^+^ questionnaire, and BP and HR were evaluated for changes in psychological and physiological responses. All sessions took place in a quiet, adjacent room under the supervision of a researcher to ensure consistency and minimize external influences.

### 2.9. Data Analysis

The software used was IBM SPSS Statistics for Windows, Version 29.0 (IBM Corp., Armonk, NY, USA); it includes built-in support for Python 3.10.4 and R 4.2.0, enhancing its capabilities to advanced statistical modeling and custom analyses.

Before performing parametric tests, the distribution of continuous variables (IDAF-4C^+^ scores, BP, HR) was assessed using both visual methods (Q–Q plots, histograms) and statistical tests (Shapiro–Wilk test). Variables meeting normality assumptions were analyzed using paired *t*-tests and one-way ANOVA. Appropriate transformations or non-parametric tests were considered for variables not normally distributed, although this was rarely required due to sample characteristics.

Descriptive statistics were employed to summarize the data, presenting frequencies for categorical variables and means or medians for continuous variables, depending on their distribution. Inferential analyses involved paired *t*-tests to evaluate within-group changes and one-way ANOVA with Tukey’s post hoc tests to assess between-group differences. Effect sizes were calculated using Cohen’s *d*, with the following interpretation thresholds applied in the context of this study: small effect ≥ 0.1, medium effect ≥ 0.3, and large effect ≥ 0.7. These thresholds were selected based on general behavioral science standards to provide clinically meaningful interpretation without universally agreed-upon dental-specific benchmarks. Cohen’s *f* was used during a priori sample size estimation to determine the number of participants required for detecting medium effects in ANOVA designs (*f* = 0.25, power = 0.80, α = 0.05); Cohen’s *d* was used post hoc to quantify pairwise effect magnitudes.

For cluster analysis, hierarchical clustering was performed using Ward’s linkage and Euclidean distance based on IDAF-4C^+^ scores, BP, HR, age, and gender. The validity of the clusters was evaluated with silhouette scores and the elbow method.

The internal consistency of the IDAF-4C^+^ questionnaire was assessed using Cronbach’s alpha, with a threshold of α ≥ 0.70 considered acceptable [27]. Cases with over 10% missing values were excluded, while those with less than 5% missing data underwent multiple imputations. Outliers were identified using standardized z-scores and Mahalanobis distance.

## 3. Results

### 3.1. Demographic Characteristics and Baseline Anxiety

A total of 189 children and adolescents participated, evenly distributed across the three groups (Table 3):

### 3.2. Within-Group Comparisons

Reductions were significant in both intervention groups, particularly in Group B (JPMR), which showed the greatest improvements in both psychological and physiological parameters (Table 4).

### 3.3. Between-Group Comparisons

The JPMR group showed significantly lower post-treatment IDAF-4C^+^ scores than BC and control. For systolic BP, only the JPMR group differed significantly from the control. Heart rate changes showed a trend (*p* = 0.053) but did not reach statistical significance between groups (Table 5).

### 3.4. Cluster Analysis: Individual Response Patterns

Hierarchical clustering using Ward’s linkage was performed on IDAF-4C^+^ scores, blood pressure (BP), heart rate (HR), age, and gender. The analysis yielded three distinct clusters. The silhouette score of 0.64 indicated good cluster separation. Table 6 summarizes each cluster’s key characteristics. Further analysis revealed clinical and demographic variables across clusters, including systolic BP and HR. Table 7 presents the summary data for each cluster.

Interpretation:

- Cluster 1 represented the most reactive group and primarily benefited from JPMR.
- Cluster 2 showed moderate improvement across interventions.
- Cluster 3 had the lowest baseline anxiety and minimal measurable benefit.

**Figure 1:**
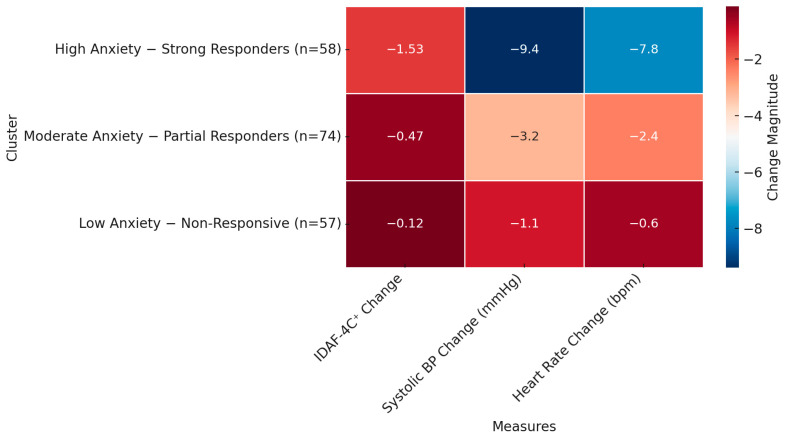
Heatmap of Standardized Change in Anxiety and Physiological Measures by Cluster.

A heatmap visualization (Figure 1) further emphasized the differential impact of interventions across clusters.

## 4. Discussion

This study assessed the efficacy of two non-pharmacological interventions—Jacobson’s Progressive Muscle Relaxation (JPM) and Breathing Control (BC)—in managing dental anxiety and physiological stress markers (blood pressure and heart rate) among children and adolescents aged 8–17. A total of 189 participants were equally allocated into three groups: JPM, BC, and standard care (control group). Using a culturally validated Romanian version of the IDAF-4C^+^ [26], alongside physiological indicators, we observed meaningful patterns of anxiety reduction and response variability across both interventions.

JPM emerged as the most effective intervention, producing a mean IDAF-4C^+^ score reduction of 1.23 points (*p* < 0.001) and a large effect size (Cohen’s *d* = 1.12), as per Cohen’s guidelines. This substantial improvement reflects JPM’s holistic impact on both somatic and cognitive–emotional dimensions of anxiety. Moreover, physiological responses confirmed these findings, with systolic blood pressure decreasing by 9.4 mmHg on average (*p* < 0.01) and moderate reductions in heart rate, suggesting a shift toward parasympathetic nervous system dominance—a hallmark of somatic de-arousal. These outcomes align with previous studies emphasizing JPM’s utility in procedural anxiety settings in children and adolescents [18,29], and they extend this evidence to a Romanian pediatric population using validated psychometric tools [26].

The BC intervention also led to significant reductions in anxiety (mean Δ = 0.64, *p* < 0.05), with a medium effect size (Cohen’s *d* = 0.61), consistent with Cohen’s classification. However, these effects were consistently smaller than those achieved through JPM.

However, these effects were consistently smaller than those achieved through JPM. While slight declines in heart rate were observed, no statistically significant changes in blood pressure were noted. This profile reflects BC’s narrower therapeutic mechanism, emphasizing autonomic modulation through respiratory control, without the full-spectrum cognitive and somatic engagement characteristic of JPM [18,19]. As such, BC may serve as a valuable intervention for children with mild anxiety profiles or in resource-limited settings where brief interventions are needed.

The control group received routine dental care without psychological intervention and did not show meaningful changes in IDAF-4C^+^ scores or physiological parameters. This underscores the limited impact of standard care on managing pre-existing dental anxiety in children and highlights the importance of integrating structured, developmentally appropriate anxiety-reduction techniques into pediatric dental practice [29].

Notably, both interventions reduced the heart rate and blood pressure, but only JPM produced statistically significant between-group differences in systolic blood pressure. Despite within-group improvements, the lack of significant group differences in the heart rate may reflect individual differences in autonomic nervous system reactivity, which can be especially pronounced in children due to developmental variability in cardiovascular regulation [14,16]. Shorter exposure times and the novelty of the intervention setting may have further influenced these physiological responses [16].

To better understand this variability, we applied hierarchical agglomerative clustering to standardized changes in psychological and physiological measures. The three-cluster solution offered nuanced, clinically relevant insights:

Cluster 1—“High anxiety—strong responders” (n = 58): This group showed the greatest reduction in IDAF-4C^+^ scores and significant physiological improvements. The predominance of younger children (8–12 years) and females in this cluster aligns with the literature, indicating heightened emotional sensitivity and greater responsiveness to relaxation techniques in these demographics [4,7]. The strong effects observed among JPM participants suggest that interventions combining cognitive and somatic components are particularly effective for children with high baseline anxiety, possibly due to enhanced engagement and the regulation of both top-down and bottom-up emotional processes [14,17].

Cluster 2—“Moderate anxiety—partial responders” (n = 74): Participants in this cluster exhibited moderate anxiety at baseline and experienced only modest psychological improvement, with no significant physiological changes. This outcome is consistent with findings that even minimal intervention or exposure to a controlled clinical setting can reduce anticipatory anxiety in children with moderate distress, potentially through habituation or increased predictability of care [1,6].

Cluster 3—“Low anxiety—non-responders” (n = 57): Children in this group maintained consistently low anxiety and showed little change in physiological or psychological indicators. These findings suggest a subgroup with higher emotional resilience and effective innate coping mechanisms [9]. For such individuals, additional psychological interventions may offer limited benefit and could be reserved for more reactive populations.

This cluster-based approach enhances our understanding of treatment effectiveness by identifying distinct psychological and physiological responses in children with anxiety. It promotes a personalized model of pediatric anxiety management, where interventions are tailored to each child’s emotional profile and developmental characteristics, rather than applied uniformly. These findings underscore the importance of individualized care strategies, demonstrating that not all children benefit equally from the same interventions; this increases the ecological validity of our clinical applications.

Previous studies have underscored the usefulness of JPM in reducing procedural anxiety in pediatric populations, especially in contexts like preoperative settings, school stress, or behavioral therapy [30,31,32,33]. Our findings are consistent while contributing novel data through the Romanian-validated version of IDAF-4C^+^ [26], addressing the limitations of previous studies that used unvalidated scales like STAI for children [34]. Other strengths include the use of exploratory clustering analysis to enhance the personalization of strategies and a controlled design that enables a clear interpretation of therapeutic effects.

This study’s findings reinforce a multi-dimensional understanding of dental anxiety, implicating physiological arousal and cognitive–emotional processing. Identifying the sources of dental anxiety (DA) is essential for developing effective mitigation strategies. Common triggers include injections and needles (trypanophobia) [35], pain [36], dental drill sounds, instruments, and even the smell of dental debris [37]. Some patients also fear choking (pseudo-dysphagia) or swallowing instruments [3,37]. The fear of pain amplifies the perception of dental procedures, often influenced by previous negative experiences or shared traumatic accounts. Even routine procedures like scaling or brushing may seem painful [38], with anxiety driven more by anticipated, rather than actual pain [39]. A systematic review confirmed a positive correlation between DA levels and fear of dental pain [40]. Dental anxiety (DA) in children is influenced by psychological and physiological factors, with temperament being a major contributor. Temperament—an early-emerging, inherited trait—modulates emotional reactivity and shapes responses to stress [41]. Children with impulsive, high-energy temperaments are more likely to resist treatment, often displaying agitation before clinical encounters [41,42], while shy children tend to internalize fear, leading to heightened DA [41,42]. Assessing a child’s communication and cooperation abilities is thus critical for successful treatment [43].

Anxiety impairs trust-building between the child and dentist, potentially triggering exaggerated emotional reactions and treatment refusal [44]. The anticipation of pain is a key driver of DA, which heightens perceived pain and reinforces the anxiety–pain cycle [45,46]. This cycle must be interrupted through targeted interventions and stress-reducing strategies.

The dentist–patient dynamic involves unequal roles: practitioners manage clinical responsibility, while patients often experience uncertainty and fear [47]. Effective communication and a calming environment foster mutual adjustment and therapeutic success [19,20]. Minimizing wait times reduces anticipatory anxiety [48], and scheduling vulnerable patients can prevent the spread of distress [49].

Creating a child-friendly dental environment can significantly influence emotional responses. Strategies like animal-shaped dental units, colorful attire, and concealing injections help reduce anticipatory fear [50,51,52]. Observing facial expressions and body language enables dentists to tailor their approach, while honest, empathetic communication fosters trust and cooperation [53].

The null hypothesis posited no significant differences among BC, JPMR, and control in reducing dental anxiety. It was rejected based on the data. BC and JPMR showed within-group reductions in IDAF-4C^+^ scores, systolic blood pressure, and heart rate (*p* < 0.05), but JPMR had the most effect. Between-group comparisons revealed significantly lower anxiety scores for JPMR than BC and control (*p* < 0.001; Cohen’s *d* = 1.12 vs. control, 0.70 vs. BC). JPMR also significantly reduced systolic BP compared to control (*p* = 0.041). Heart rate differences showed a trend (*p* = 0.053) but were not significant. Cluster analysis supported these findings, identifying a subgroup with high anxiety and a strong response to JPMR. These results demonstrate that JPMR was more effective than BC and control, justifying rejection of H_0_.

The clinical implications are substantial. Implementing brief relaxation interventions, especially JPM, before dental procedures may significantly reduce patient distress, increase cooperation, and improve procedural efficiency in pediatric dental practices. These methods are non-invasive, cost-effective, and easily integrated into routine care. Treatment response variability underscores the necessity for pre-treatment screening tools, improved by decision algorithms that align interventions with patient profiles based on factors such as baseline anxiety, age, sex, or dental history [4,18,31,36,48].

Given its minimal training requirements, non-invasiveness, and low cost, JPM represents a practical and effective strategy for pediatric dental anxiety. Its integration into routine care could improve both patient experience and procedural cooperation.

Despite its strengths—including controlled group allocation, a culturally validated anxiety measure, and objective physiological data—this study has limitations. The short-term design precludes conclusions about long-term anxiety reduction or behavioral changes. The fidelity of intervention delivery was not formally assessed, potentially introducing variability in therapeutic exposure. Additionally, contextual factors such as parental anxiety, previous trauma, or clinician–child rapport were not measured, yet these could substantially influence outcomes. Physiological data (HR and BP) may also be sensitive to external stressors unrelated to dental anxiety. Finally, while the cluster analysis yielded insights, it was exploratory and lacked external validation; replication in larger samples is necessary.

Future research should employ longitudinal follow-up to assess the durability of anxiety reduction and examine its impact on future dental cooperation. Personalized interventions need the investigation of behavioral predictors, such as temperament, attachment style, or family dynamics. Furthermore, the digital integration of JPM via mobile applications could enable pre-visit training, increase accessibility, and prepare children outside the clinical setting. Expanding this model to multi-center trials would improve generalizability and allow cross-cultural comparisons, especially in underserved populations.

## 5. Conclusions

This study affirms Jacobson’s Progressive Muscle Relaxation as a superior intervention for reducing dental anxiety in pediatric patients, offering psychological relief and physiological regulation. While Breathing Control shows partial benefits, its effects are less consistent and generally milder. Response variability, captured through cluster analysis, highlights the value of individualized approaches in anxiety management. Incorporating psychological techniques like JPM into standard pediatric dental practice holds promise for improving treatment outcomes, patient experience, and clinical efficiency.

## Figures and Tables

**Table 1:** Blood pressure average values.

Age	Systolic BP (mm Hg)	Diastolic BP (mm Hg)	Status
10 years	102 ± 11	64 ± 10	Normal
13 years	108 ± 13	66 ± 11	Normal
16 years	115 ± 12	67 ± 11	Normal

**Table 2:** Heart rate values in children and adolescents by age and sex.

Age (Years)	Sex	Mean HR (Beats/Min)	Normal Range (Beats/Min)
10	Girls and Boys	90	70–110
12	Girls	90	70–110
12	Boys	85	65–105
14	Girls	85	65–105
14	Boys	80	60–100
16	Girls	80	60–100
16	Boys	75	55–95
18	Girls	75	55–95
18	Boys	70	50–90

**Table 3:** Demographic characteristics and baseline anxiety.

Variable	Group A (BC)	Group B (JPMR)	Control	*p*-Value
Age (years, mean ± SD)	12.4 ± 2.5	12.6 ± 2.4	12.5 ± 2.6	0.88
Gender (M/F)	34/29	32/31	33/30	0.94
Baseline IDAF-4C^+^	3.32 ± 0.41	3.33 ± 0.44	3.31 ± 0.40	0.92

No significant differences observed at baseline. Abbreviations: IDAF-4C^+^ = Index of Dental Anxiety and Fear Core Module.

**Table 4:** Pre- and post-intervention measures (paired *t*-tests).

Measure	Time	Group A (BC)	Group B (JPMR)	Control	*p*-Value (Within Group)
IDAF-4C^+^	Pre	3.32 ± 0.41	3.33 ± 0.44	3.31 ± 0.40	—
	Post	3.02 ± 0.43 ^1^	2.64 ± 0.39 ^2^	3.28 ± 0.38	<0.001
Systolic BP (mmHg)	Pre	108.9 ± 7.8	109.1 ± 7.5	108.4 ± 8.0	—
	Post	105.7 ± 7.2 ^1^	102.2 ± 6.9 ^2^	107.6 ± 7.9	0.038
Heart Rate (bpm)	Pre	91.0 ± 9.3	91.5 ± 8.8	90.7 ± 9.1	—
	Post	87.6 ± 8.6 ^1^	83.1 ± 8.1 ^2^	90.2 ± 9.3	0.041

^1^ *p* < 0.05, ^2^ *p* < 0.01 (within-group, pre-post comparison using paired *t*-tests). Abbreviations: IDAF-4C^+^ = Index of Dental Anxiety and Fear; BP = blood pressure; and bpm = beats per minute.

**Table 5:** Post-intervention outcomes (ANOVA and effect sizes).

Outcome	ANOVA *p*-Value	Significant Post Hoc Differences	Cohen’s *d* (Effect Size)
IDAF-4C^+^ Score	<0.001	JPMR < BC < Control	JPMR vs. Control = 1.12
			JPMR vs. BC = 0.70
Systolic BP	0.041	JPMR < Control	JPMR vs. Control = 0.62
Heart Rate	0.053 (trend)	No significant differences	—

Effect sizes (Cohen’s *d*) are interpreted according to standard guidelines as small ≥ 0.1, medium ≥ 0.3, large ≥ 0.7; values reported here fall within the medium-to-large range [27]. JPMR produces the greatest reductions in dental anxiety scores (IDAF-4C^+^: Δ = −1.23, 95% CI: −1.36 to −1.10, *p* < 0.001; Cohen’s *d* = 1.12, 95% CI: 0.89–1.34), followed by BC (Δ = −0.64, 95% CI: −0.79 to −0.48, *p* < 0.05; Cohen’s *d* = 0.61, 95% CI: 0.39–0.83).

**Table 6:** Cluster characteristics.

Cluster	Key Traits	n	Predominant Group
Cluster 1	High anxiety, strong reduction with JPMR, younger, more females	58	JPMR
Cluster 2	Moderate anxiety, partial responders	74	Mixed
Cluster 3	Low anxiety, minimal response	57	Control and BC

Abbreviations: BC = Breathing Control; and JPMR = Jacobson’s Progressive Muscle Relaxation.

**Table 7:** Cluster summary data.

y	Baseline IDAF	Post IDAF	Δ Systolic BP	Δ HR	Age Range	Gender Trend
Cluster 1	3.91	2.38	–9.4 mmHg	–6.5 bpm	8–12 years	More females
Cluster 2	2.91	2.54	–3.2 mmHg	–2.4 bpm	10–14 years	Balanced
Cluster 3	2.34	2.30	–0.8 mmHg	–0.5 bpm	13–17 years	Balanced

Abbreviations: IDAF = Index of Dental Anxiety and Fear; BP = blood pressure; HR = heart rate; and Δ = change from pre to post.

**Figure A1:**
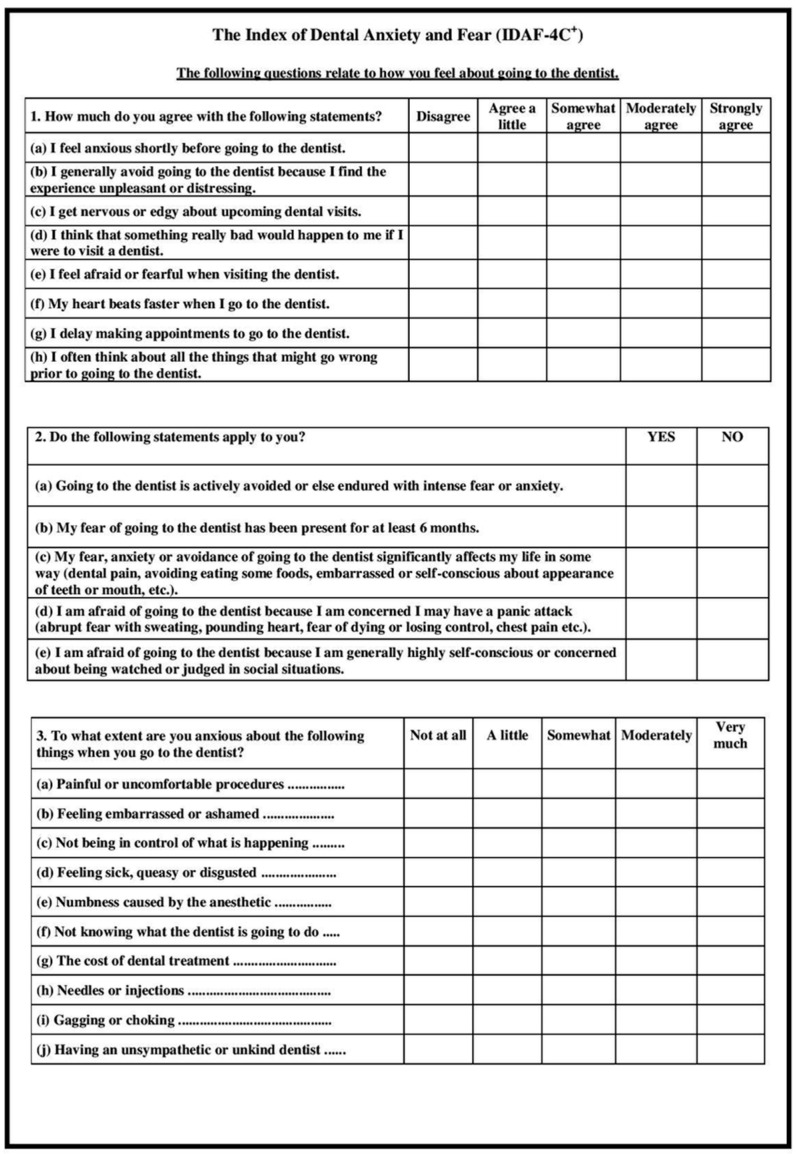
The Index of Dental Anxiety and Fear (IDAF-4C^+^).

## Data Availability

Data supporting reported results can be found by emailing Sorana Maria Bucur at bucursoranamaria@gmail.com.

## Appendix A

### Appendix A.1. Jacobson’s Progressive Muscle Relaxation (JPMR) Technique in the Dental Setting [3]

Progressive Muscle Relaxation (PMR), developed by Edmund Jacobson, can be effectively adapted for children and adolescents in a dental environment to ease anxiety and enhance cooperation during treatment. This method systematically involves tightening and releasing specific muscle groups, fostering an awareness of tension and relaxation. The goal is to induce a state of calm before or during dental care, especially in anxious patients.

The technique is modified for comfort and feasibility and applied in the dental chair. The child is guided step-by-step, in a calm voice, to gently contract and relax various muscle groups while remaining seated or reclined. A relaxed position in the dental chair is encouraged: feet flat, arms resting loosely, and head supported. Relaxing background music can further assist the process.

Guided Steps Adapted for the Dental Chair:

1. Begin with a few slow, gentle breaths. Ask the patient to inhale softly, pause, and then exhale slowly.
2. Feet and legs: The child pulls the toes upward slightly toward the knees, just enough to feel the muscles engage, then holds for a moment, and releases. Then, the child presses the heels down onto the footrest, holds briefly, and relaxes.
3. Legs and hips: Ask the child to press their knees gently together, hold briefly, and allow them to drift apart slightly. Then, have them squeeze the gluteal muscles together, hold, and relax.
4. Abdomen: Guide the child to draw their tummy slightly toward their spine, hold, and then release. Emphasize noticing how their body feels when relaxed.
5. Shoulders and arms: Ask the child to slowly lift their shoulders toward their ears, just enough to notice tension, hold, and let them drop. Then, have them gently press their elbows into their sides, hold, and relax.
6. Hands: Instruct them to softly clench their fists, hold for a few seconds, then open their hands, and let them rest.
7. Neck and face: Tilt the head forward, make a break, and let it return to a natural, balanced position. Ask them to clench their teeth slightly, hold, and then relax the jaw. Press lips together gently, hold, and then let them loosen naturally. Instruct the child to press their tongue against the roof of their palate, hold it, and let it fall into a relaxed position.
8. Eyes and forehead: Ask the child to squint the eyes gently and then release them. Finally, have them furrow their brow slightly, hold, and then allow the forehead to soften.

Throughout the process, the clinician or assistant can softly prompt the child to notice the difference between tension and relaxation. The technique typically takes 10–15 min but can be shortened based on clinical needs or the patient’s age and attention span.

This adaptation of JPM has been shown to help reduce dental anxiety in children and enhance cooperation, creating a positive clinical experience for both the patient and the practitioner.
